# Brick tea consumption and its relationship with fluorosis in Tibetan areas

**DOI:** 10.3389/fnut.2022.1030344

**Published:** 2022-12-13

**Authors:** Cai Wen, Qing Zhang, Fei Xie, Jixin Jiang

**Affiliations:** ^1^Department of Oral Implantology, Affiliated Stomatological Hospital of Southwest Medical University, Luzhou, Sichuan, China; ^2^Department of VIP Dental Service, Affiliated Stomatological Hospital of Southwest Medical University, Luzhou, Sichuan, China; ^3^Luzhou Key Laboratory of Oral and Maxillofacial Reconstruction and Regeneration, Affiliated Stomatological Hospital of Southwest Medical University, Luzhou, Sichuan, China; ^4^Department of Nosocomial Infection Control, Affiliated Hospital of Southwest Medical University, Luzhou, Sichuan, China; ^5^School of Stomatology, Southwest Medical University, Luzhou, Sichuan, China

**Keywords:** brick tea-type fluorosis, brick tea, dental fluorosis, skeletal fluorosis, Tibetan, food security

## Abstract

Brick tea-type fluorosis (BTF) due to a high intake of brick tea is possible in Tibetan populations, and dental fluorosis (DF) and skeletal fluorosis (SF) are its primary manifestations. To determine the prevalence of DF and SF and their relationships with brick tea intake in Tibetan populations, a literature review was conducted for studies published between 1994 and 2021. The available evidence revealed that brick tea may be produced from older stems and leaves of the tea plant and that the fluoride content of brick tea exceeds the national standard. The harsh environment of the plateau has led to limited food sources for the local Tibetan people who form the habit of drinking tea leaves as a satiation solution to digest greasy food and replenish vitamins, and regular consumption of brick tea leads to excessive exposure of Tibetan residents to fluoride. Studies in Tibet showed that the prevalence of DF in children was 14.06–75.93% in different districts, and the overall pooled prevalence of DF was 26.08%. The prevalence of SF in adults was 19.90–74.77% in different Tibetan districts, and the overall pooled prevalence of SF was 33.84%. The analysis of risk factors showed that the prevalence of BTF may be related to high-altitude and different working and living conditions, and BTF in children may be associated with fluoride intake during mothers’ pregnancy and lactation. With the development of bioinformatics research, gene polymorphisms were suspected to be related to susceptibility to fluorosis in Tibetan populations. The study of BTF in Tibetan people needs to be further investigated and standardized, and additional studies evaluating the pathogenesis and preventive measures of BTF are warranted.

## Introduction

Fluorosis is an endemic disease that is closely associated with the excessive intake of fluoride. Fluorine ranks 24th among all elements in universal abundance and 13th in terrestrial abundance, accounting for 0.08% of the Earth’s crust (1). An appropriate intake of fluoride is beneficial to health (2). Fluoride participates in the metabolism of calcium and phosphorus and improves the strength of teeth and bones (3, 4). However, fluoride overdose may cause detrimental health effects, and fluoride can be ingested through drinking water, fluoride supplementation, or fluoridated toothpaste. According to the World Health Organization, long-term fluoride intoxication is associated with damage to the teeth and bones as well as the endocrine, gastrointestinal, renal, neurological, and reproductive systems (5, 6). Dental fluorosis (DF) and skeletal fluorosis (SF) are the most common manifestations of excessive fluoride intake (7–9). Symptoms of DF include cloudy white or chalky lines, spots, or marks on teeth, yellow or brown discoloration, surface irregularities, and visible pits in tooth enamel. SF is a metabolic bone disease, and it is the most common and severe manifestation of fluorosis in adults. The main clinical presentations of SF include lumbar and leg joint pain, joint rigidity, bone deformation, and spinal cord compression.

Endemic fluorosis is prevalent in China because of the high-fluoride content of water sources and coal combustion in some areas (10). The fourth National Oral Health Survey conducted in mainland China in 2016 (11) revealed that DF had a prevalence of 40.3% in Tibet, which was the third highest after those of Guizhou Province and Tianjin City. The fluoride intake of Tibetan people is associated with certain local factors, and the excessive intake of brick tea may be one of them.

The tea plant is a perennial plant that can maintain a steady yield for up to 100 years. It prefers to grow in acidic soil (pH 3.5–5.6) where it can obtain the element fluorine. After the decomposition of the aluminum fluoride complex in the soil, fluoride in ionic form (F^–^) is absorbed by the roots, and excessive amounts of F^–^ form complexes with Al^3+^ and are transferred to old leaves (12, 13). Tea beverages are popular in many regions worldwide, and the fluoride enrichment effect of the tea plant carries a risk of excessive fluoride intake for tea drinkers (14–19). The diversity of tea beverages, various production techniques, and soil conditions of tea-producing areas may result in varying differences in the fluoride content of tea (14, 16–18, 20–26).

Since ancient times, Chinese people have had the tradition of drinking tea. The types of tea and drinking habits differ across regions. Tibetans are primarily distributed in the Qinghai–Tibet Plateau, which has an average altitude of 3,000–5,000 m. Owing to the cold weather, high-altitude, and higher radiation exposure, the residents of this region are accustomed to eating high-fat and high-sugar foods to adapt to the harsh environment. Tea consumption is important for digesting greasy food and replenishing vitamins. However, Tibetan regions with high-altitude mountain landforms do not produce tea; thus, the supply of tea is primarily from low-altitude Han-inhabited areas (27). The ancient Tea Horse Road (28, 29), a famous passage for economic and cultural exchanges in ancient China that rose from the Tang Dynasty (approximately 8th century A.D.), was derived from the complementary trade of tea and horses between the Tibetan and Han people.

**FIGURE 1:**
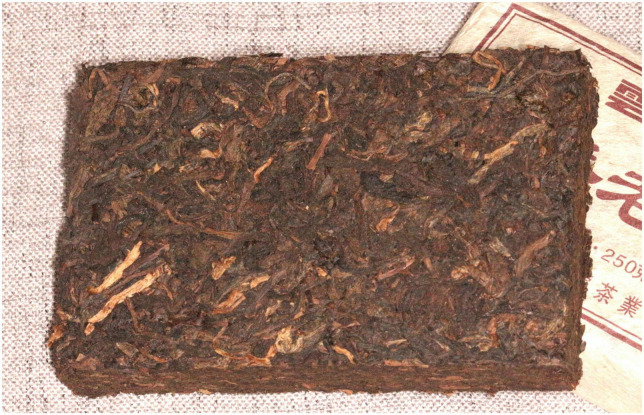
A piece of brick tea produced in Yunnan province. Brick tea looks like a brick, and is a representative type of pressed tea, which is made of tea leaves, tea stems, and sometimes tea dust.

As tea plants selectively absorb fluoride from the soil, the concentration of fluoride increases with maturity (30, 31). The materials used to make brick tea include old leaves and stems from the tea plant, and as the name suggests, this tea is molded into a brick shape for better preservation (Figure 1). Therefore, drinking brick tea in large quantities may increase fluoride intake compared to consuming other drinks, potentially resulting in brick tea-type fluorosis (BTF).

Existing studies (27, 32–36) have reported a relationship between brick tea consumption and fluorosis in Tibet. These studies were conducted in isolated cities, towns, or villages, and the information was relatively limited. To comprehensively compare fluoride intake from brick tea, prevent the occurrence of fluorosis, and confirm the relationship between brick tea consumption and fluorosis, the factors associated with excessive fluoride intake need further examination. A review is warranted to assess the prevalence of BTF and evaluate the effectiveness of the prevention and control of endemic BTF in recent years. In this study, the pooled prevalence rates of DF and SF and their existing risk factors were assessed to minimize bias and ensure objectivity, authenticity, and reliability of the research results, and a literature review was conducted to identify possible risk factors.

## Materials and methods

### Search strategy and identification criteria

Reasonable inclusion and exclusion criteria were established by evaluating the research content and scope of previous reports on BTF. Although many reports about brick tea are reported in Chinese, to ensure the objectivity, openness, and repeatability of this study, only articles written in English were included. The electronic search databases of PubMed and Cochrane Library were searched for studies published between January 1994 and September 2021. The search terms were “(“brick tea” OR “tea” OR “brick-tea”) AND (“fluorosis” OR “dental fluorosis” OR “skeletal fluorosis” OR “fluoride”).” The reference lists of the included articles were added to the screening list to identify eligible studies. Two authors independently screened the titles and abstracts of these studies and retrieved the full texts of potentially eligible articles if they met the inclusion criteria. Any conflicts regarding the inclusion or exclusion of a study were discussed, and a third researcher was invited to make the final judgment.

### Inclusion and exclusion criteria

To meet the requirements of the analysis and reduce bias, studies were required to meet the following criteria: (1) The study was conducted in Tibetan residential areas, i.e., the Tibet Autonomous Region, the Qinghai Province, and portions of the Sichuan and Yunnan provinces; (2) the included participants were local individuals from the field sites rather than selected volunteers; (3) clear diagnostic criteria for fluorosis and accurate fluoride concentration measurement methods were reported; and (4) the time of the survey was reported. In cases of multiple reports of the same samples in the same area during the same period of time, the report with the most detailed information was included.

The exclusion criteria included duplicate articles; articles that discussed fluorosis of animals or laboratory research; articles focusing on fluoride intoxication that was not caused by brick tea; articles focusing on the association of fluoride with other health-related issues, such as cancer, intelligence, or physical development; studies not conducted on Tibetan individuals or in Tibetan-inhabited areas; and studies with unclear age, sex, and other demographic data of the surveyed participants or data that could not be obtained from the authors.

### Literature screening, data extraction, and compilation

Relevant data were subsequently extracted and used to compile a database. The data from all included studies were clearly tabulated. Deviations were considered and identified during the quality assessment stage. The following data were collected from each study using specifically designed tables: Author; year of publication; time and place of investigation; altitude of the investigated place; sample size; demographic information; sex; occupation; volume of daily brick tea consumption; prevalence of DF and SF of the investigated participants; fluoride level of brick tea; and fluoride level of brick tea infusions. The altitude was based on the average height of the urban area where the study was located according to publicly available information, like Baidu Baike, if the data was not given in article.

The fluoride level of brick tea was the enumeration of the fluoride content of dry brick tea obtained from different studies, and the volume of daily brick tea consumption was based on the amount of brick tea consumed or the amount of liquid applied. Due to the different methods reported in various studies, the fluoride and tea consumption levels may use different units of measurement; this study enumerated them without changing the units. The prevalence of DF and SF were based on the ratio of the number of patients with fluorosis in the study to the total number of enrolled people in the study. The DF index was reported by the authors of the study if available. Based on these data, the overall pooled prevalence of DF and SF were calculated. Pearson’s correlation coefficients of the relationship between the prevalence of DF and SF and the altitudes of the investigated areas were also calculated.

## Results

### General database statistics

The initial electronic and manual searches yielded 297 articles. After screening the titles and abstracts, 274 articles were excluded because they were not human studies, were not conducted in Tibetan areas, did not focus on brick tea consumption, did not relate to DF and SF, or were not available in English. Then, 23 articles were read and reviewed in full. Among them, eight articles (37–44) were excluded because they had unclear demographic data or unclear SF or DF results, or the results were not consistent with the theme. Finally, 15 studies were included in the research: 6 studies (27, 35, 36, 45–47) that focused on BTF in Tibetan-inhabited areas were included in group 1; 4 studies (27, 45, 47, 48) that discussed the fluoride content of brick tea were included in group 2 (three articles were included in both group 1 and 2); and 8 articles (38, 43, 49–54) were included in group 3 because they primarily discussed the genetic susceptibility of Tibetans to fluorosis.

The PRISMA flowchart of the literature screening process was constructed (Supplementary material). Table 1 provides a summary of articles related to BTF prevalence. DF was investigated only in children in most included studies; however, the study by Fan et al. (45) studied DF in both children and their mothers. SF was only studied in adults in all included literature.

**TABLE 1:** Included studies on brick tea-type fluorosis.

References	Districts	Regions	Participants	Diseases
Cao et al. (35)	Daofu	Tibet AR	Children and adults	DF and SF
Cao et al. (36)	Xiege’er	Tibet AR	Children	DF
	Zhangmu	Tibet AR		
Cao et al. (27)	Naqu	Tibet AR	Adults	SF
Fan et al. (45)	Lhasa	Tibet AR	Children and adults	DF and SF
	Ali	Tibet AR		
	Nagqu	Tibet AR		
	Shigatse	Tibet AR		
	Shannan	Tibet AR		
	Linzhi	Tibet AR		
	Changdu	Tibet AR		
Zhang et al. (46)	Ganzi	Sichuan	Children and mothers	DF
Li and Zhao (47)	Haidong	Qinghai	Children	DF
	Hainan			
	Yushu			

AR, Autonomous Region; DF, dental fluorosis; SF, skeletal fluorosis.

### Fluoride level of brick tea

The Chinese national standard requires the fluoride concentration of dry tea to be within 300 mg/kg (GB 19,965-2005). Four articles disclosed the fluoride content of dry brick tea. Table 2 provides a summary of the fluoride content analysis. Three studies (27, 47, 48) compared the fluoride content of dry tea leaves in different sampling districts, whereas the researchers of one article (45) disclosed the fluoride content of tea infusions. Among the dry brick tea samples from Tibet, all samples had fluoride levels exceeding the national standard (i.e., 348.34–1085.70 mg/kg), except for one sample from Shigatse, which had a fluoride level of 96.53 mg/kg. The lowest reported fluoride content occurred in Shigatse (3.74 ± 5.09 mg/L), while the highest reported fluoride content occurred in Nagqu (10.90 ± 1.90 mg/L). Because the fluoride content of tea infusions is directly related to the method of mixing and there is no national standard for fluoride in beverages, it was impossible to conclude whether the fluoride content in tea beverage was significantly over the limit or not for studies that only evaluated tea solutions.

**TABLE 2:** Included studies on the fluoride concentration of brick tea.

References	Districts^1^	Region	Tea-type	Content
Cao et al. (48)	Bianxiao	Sichuan and Hunan	Dry brick tea	491.8 mg/kg
Cao et al. (27)	Naqu	Tibet AR	Dry brick tea	739 ± 27 mg/kg
Fan et al. (45)	Lhasa	Tibet AR	Brick tea infusion	8.15 ± 2.33 mg/L
	Ali	Tibet AR		7.38 ± 6.20 mg/L
	Nagqu	Tibet AR		10.90 ± 1.90 mg/L
	Shigatse	Tibet AR		3.74 ± 5.09 mg/L
	Shannan	Tibet AR		8.65 ± 2.84 mg/L
	Linzhi	Tibet AR		5.34 ± 4.06 mg/L
	Changdu	Tibet AR		4.66 ± 0.67 mg/L
Li and Zhao (47)	Haidong	Qinghai	Dry brick tea	724.74 ± 322.25 mg/kg
	Hainan	Qinghai		882.37 ± 404.71 mg/kg
	Yushu	Qinghai		1467.00 ± 279.20 mg/kg

AR, Autonomous Region.

^1^With the exception of Bianxiao, the other districts in this table refer to the places where the tea samples were obtained rather than where the tea was produced.

### Prevalence of brick tea-type DF among children

In the five studies that included the prevalence of DF, 13,127 children were examined and 2,404 children were thought to have developed some degree of DF. The reported prevalence rate of DF was 14.06–75.93%, and the pooled prevalence rate of DF was 26.08%. The areas with the highest DF prevalence rates were Xiege’er (75.93%) and Ganzi (62.23%). Dean’s DF index was used in most studies (36, 46, 47), and the Chinese national standard of DF was used in one study (45) (Table 3). According to our detailed examination of the statements of the two standards, the classification and statements of both are the same. The highest DF index was reported in Xiege’er (3.11), while the lowest index was reported in Hainan Autonomous Prefecture (0.24). However, it is important to note that differences may be due to the possible detection heterogeneity of different studies.

**TABLE 3:** Prevalence of dental fluorosis in different studies.

District	Altitude (m)	Index for measurement of DF	*N*	Prevalence of DF (%)	DF index
Zhangmu (36)	2,000	Dean’s	116	25.86	0.67
Xiege’er (36)	4,300	Dean’s	403	75.93	3.11
Nagqu (45)	4,500	National criteria (WS/T 208-2011)^2^	63	50.79	1.75
Lahsa (45)	3,650	National criteria (WS/T 208-2011)^2^	87	44.83	1.79
Shigatse (45)	4,000	National criteria (WS/T 208-2011)^2^	118	27.97	1.61
Shannan (45)	3,700	National criteria (WS/T 208-2011)^2^	109	25.69	2.07
Linzhi (45)	3,100	National criteria (WS/T 208-2011)^2^	13	23.08	1.17
Changdu (45)	3,500	National criteria (WS/T 208-2011)^2^	33	21.21	2.57
Daofu (35)	3,000	Dean’s	375	51.2	1.33
Ganzi (46)	2,600	Dean’s	368	62.23	2.30
Haidong (47)	N/A 2,000^1^	Dean’s	2,677	15.09	0.26
Hainan (47)	N/A 3,000^1^	Dean’s	3,975	14.06	0.24
Yushu (47)	N/A 4,000^1^	Dean’s	4,790	32.61	0.55
Sum			13,127	26.08%	

DF, dental fluorosis; N/A, not available.

^1^The altitudes of these areas are not available from the literature, but they are available from publicly available information.

^2^Study by Fan et al. (45) used the national criteria in the text instead of Dean’s index, but the two have very similar presentation and scoring criteria.

### Prevalence of brick tea-type SF among adults

Three articles (27, 35, 45) discussed the prevalence rate of SF. A total of 2,089 adults were examined in these studies, and 923 adults were detected to have some degree of SF. The reported prevalence of SF was 19.90–74.77%, and the pooled prevalence rate of SF was 33.84%. The districts with the highest SF prevalence were Ali (74.77%) and Nagqu (68.25%). SF had no well-recognized index; therefore, the index of SF was not calculated (Table 4). Since there is no internationally recognized evaluation index for SF, no indexing score was given, but the authors classified them according to severity.

**TABLE 4:** Prevalence of skeletal fluorosis in different studies.

District	Altitude (m)	Index for measurement of SF	*N*	Prevalence of SF (%)
Nagqu (45)	4,500	National criteria (WS/T 192-2008)	315	68.25
Lahsa (45)	3,650	National criteria (WS/T 192-2008)	115	59.13
Shigatse (45)	4,000	National criteria (WS/T 192-2008)	124	35.48
Shannan (45)	3,700	National criteria (WS/T 192-2008)	322	27.64
Linzhi (45)	3,100	National criteria (WS/T 192-2008)	201	19.90
Changdu (45)	3,500	National criteria (WS/T 192-2008)	136	52.94
Ali (45)	4,500	National criteria (WS/T 192-2008)	107	74.77
Daofu (35)	3,000	<Manual of preventing endemic fluorosis>	658	32.83
Naqu (27)	4,500	<Standards of endemic fluorosis>	111	89.0
Sum			2,089	33.84%

SF, skeletal fluorosis.

### Fluoride intake per day for included participants

Fluoride intake among Tibetans may come from various sources, such as brick tea, zanba (a type of food made from cooked barley), butter tea, and water. Table 3 shows the average total daily fluoride intake per person from brick tea in different districts for the included participants. Fluoride intake differed significantly between districts and was much higher than the national standard in most areas (National standard GB 17,018-2011: The daily fluoride intake should be <3.5 mg per person). Among the four studies that reported daily fluoride intake, only Cao et al. (36) reported a fluoride intake of 2.905 mg/day in Zhangmu, which did not exceed the standard. Fan et al. (45) reported that the highest fluoride intakes were in Naqu (55.630 mg/day) and Ali (35.60 mg/day) in their study (Table 5).

**TABLE 5:** Fluoride intake through brick tea in different studies.

District	Region	Altitude (m)	Daily fluoride intake from brick tea (mg/day)
Zhangmu (36)	Tibet AR	2,000	2.905^1^
Xiege’er (36)	Tibet AR	4,300	8.045^1^
Nagqu (45)	Tibet AR	4,500	55.630
Lahsa (45)	Tibet AR	3,650	25.026
Shigatse (45)	Tibet AR	4,000	9.800
Shannan (45)	Tibet AR	3,700	22.350
Linzhi (45)	Tibet AR	3,100	12.010
Changdu (45)	Tibet AR	3,500	23.430
Ali (45)	Tibet AR	4,500	35.600
Daofu (35)	Tibet AR	3,000	7.80^2^
			3.89^3^
Naqu (27)	Tibet AR	4,500	8.03^4^

AR, Autonomous Region.

^1^Data for children.

^2^Data for individuals aged >15 years.

^3^Data for individuals aged 8–15 years.

^4^Data for adults.

### Correlations of DF and SF with altitude

Pearson’s correlation coefficients of the relationship between the prevalence of DF and SF and the altitudes of the surveyed areas were 0.389 and 0.803, respectively. Moreover, Pearson’s correlation coefficient of the DF index with the altitude was 0.5157, and that of the daily fluoride intake from brick tea with the altitude was 0.5186. A significant difference in Pearson’s correlation coefficient was only detected between the prevalence of SF and the altitude (Table 6).

**TABLE 6:** Pearson correlation coefficients of DF- and SF-related variables with altitude.

	*r*	95% confidence interval	*P* (two-tailed)
Prevalence of DF%	0.3894	−0.2057 to 0.7743	0.1884
Prevalence of SF%	0.8030	0.2976 to 0.9568	0.0092*
Dean’s index of DF	0.5157	−0.04924 to 0.8307	0.0712
Daily fluoride intake from brick tea^1^	0.5186	−0.2220 to 0.8797	0.1526

^1^Daily fluoride intake data from children in Zhangmu, Xiege’er, and Daofu were excluded. *Indicates a statistically significant difference.

### Relationship between fluorosis susceptibility and genotype

In this study, eight articles (38, 49–55) related to the genetic susceptibility to fluorosis were obtained (Table 7). Wu et al. (55) investigated the role of *GSTP1* rs1695 polymorphisms in the susceptibility to fluorosis. Pei et al. (51) reported a significant correlation between the *MMP2* rs2287074 genotype and SF severity, and the A allele of *MMP2* rs2287074 was a protective factor in Tibetans. Yang et al. (50) indicated that Tibetans were more likely to develop moderate and severe brick tea-type SF, and CT/TT genotypes of vitamin D receptor-*Fok*I may be a protective factor. Li et al. (54) indicated that a polymorphism in the extrapituitary prolactin promoter may decrease the risk of brick tea-induced SF in the Kazakh people. Chu et al. (38) suggested an association between the *AlOX15* gene polymorphism and SF risk in Han participants. The CC/CC diplotype had a protective effect on SF risk in Han participants, whereas the CA/CC diplotype influenced SF risk in participants aged ≥65 years. Liu et al. (53) indicated that serum Klotho may be a potential mediator of SF in BTF-endemic areas. Yang et al. (49) suggested there might be differential genetic influence on SF risk in Kazakh and Tibetan participants and that this difference might be modified by tea fluoride intake. However, a study by Lou et al. (52) did not find an association between *BMP2* single nucleotide polymorphisms and SF in their cross-sectional case-control study.

**TABLE 7:** Genetic studies associated with brick tea-type fluorosis susceptibility.

References	Country	Gene	Population	District
Wu et al. (55)	China	Glutathione S-Transferase pi-1	Tibetan, Mongolian, Han, Kazakh, and Russian	Qinghai, XUAR, Inner Mongolia
Yang et al. (50)	China	Vitamin D Receptor Gene FokI	Tibetan, Kazakh, Mongolian, and Han	Qinghai, XUAR, Inner Mongolia
Li et al. (54)	China	Prolactin Promoter at -1,149	Tibetan, Kazakh, and Mongolian	Qinghai, XUAR, Inner Mongolia
Pei et al. (51)	China	Matrix Metallopeptidase-2	Tibetan and Kazakh	Qinghai, XUAR
Yang et al. (49)	China	Frizzled Related Protein 1	Tibetan and Kazakh	Qinghai, XUAR
Liu et al. (53)	China	Serum Soluble Klotho	Tibetan and Kazakh	Qinghai, XUAR
Lou et al. (52)	China	Bone Morphogenetic Protein 2	Tibetan and Kazakh	Qinghai, XUAR
Chu et al. (38)	China	Arachidonate-15-Lipoxygenase	Tibetan, Kazakh, and Han	Qinghai, Inner Mongolia, XUAR

XUAR, Xinjiang Uyghur Autonomous Region.

**FIGURE 2:**
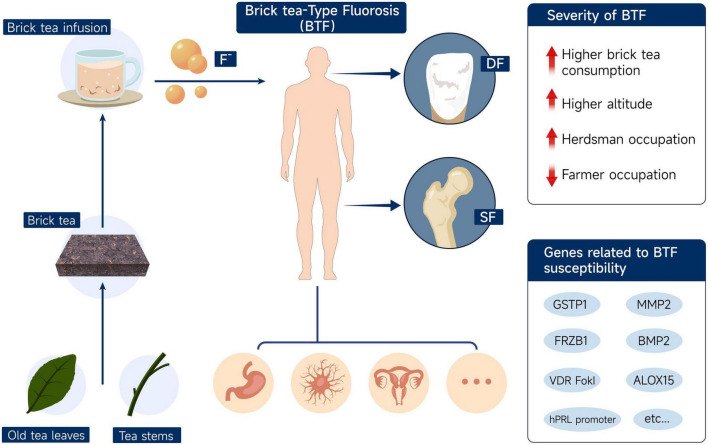
Schematic diagram of fluoride accumulation in brick tea, the manifestations of brick tea-type fluorosis, and possible influencing factors. DF, dental fluorosis; SF, skeletal fluorosis; BTF, brick tea-type fluorosis.

A schematic diagram (Figure 2) shows the fluoride accumulation effect of brick tea in old leaves and stems, the result of BTF in Tibetan populations, and possible influencing factors.

## Discussion

### Relationship between brick tea and fluoride

Because of the accumulation effect of fluoride in tea plants, drinking tea has become a source of excessive fluoride in the human body. The influences of different tea-producing areas, production methods, drinking habits, and dosages on the development of fluorosis should be noted.

Chandrajith et al. (16) reported that in Sri Lanka, the traditional habits of locally blended black tea consumption can cause an additional intake of fluoride and promote adverse health conditions that may also be related to chronic kidney disease. Whyte et al. (17) reported that the popular instant tea powder contributes to approximately 80% of F^–^ exposure, and SF from the habitual consumption of large volumes of extra-strength instant tea calls for recognition. Apart from these, several studies (14, 56–60) have also raised health concerns regarding the excessive intake of fluoride from tea in certain regions.

Brick tea is relatively low-end tea, especially that sold in remote mountainous areas. Because of the long distances traveled, tea needed to be preserved for a long time and easily transported, so it was often pressed into the brick form that gave it its name. The ethnic people in the mountainous areas consume more meat and milk and less vegetables, and drinking tea not only helps digest greasy food, but it also supplements essential vitamins and trace elements. Therefore, it is said that “it is better to go without food for a day than without tea for a day.” Brick tea has become a necessity of Tibetan life.

Previous studies have reported that the fluoride content of brick tea was higher than that of ordinary tea (44, 48, 61). The Chinese national standard requires that the fluoride concentration of dry tea should be within 300 mg/kg. Studies (27, 47, 48) on BTF in Tibet have reported that the fluoride content of dry brick tea samples greatly exceeded the national standard, some of which reached three to five times the limit. In addition, animal experiments (47, 48) have also shown that rats exposed to brick tea infusions present with DF. Therefore, the association between high-fluoride brick tea consumption and endemic fluorosis in Tibet can be verified.

To distinguish the link between brick tea and fluorosis, we also need to know if the source of fluoride comes from other substances used in tea brewing, such as water and fuel. However, the fluoride content of drinking water in nearly all Tibetan residential areas is within the national drinking water range (≤1.2 mg/L, GB 5,749-2006), and the possibility of water-initiated fluorosis is low (27, 36, 39, 45). Among the four studies that reported fluoride concentrations in local drinking water samples, only the concentration of one water sample exceeded the national standard (in Ali, 2.01 mg/L). The fluoride concentrations of other water samples were lower than the national standard (0.03–0.96 mg/L). Moreover, the use of coal is often a cause of fluorosis, but Tibetan-inhabited areas are not coal-producing areas and do not have the tradition of using coal. Tibetan individuals generally use wood and cow dung as fuel in the countryside. Therefore, the possibilities of fluoride intake caused by coal pollution and drinking water can be eliminated.

### Relationship between DF and brick tea

Dental fluorosis is characterized by chalky-white to brown patches on the enamel and, in severe cases, defects of the enamel. Fluorosis can be divided into three types based on severity: chalky type (mild), discoloration type (moderate), and defect type (severe) (62). DF is more common in permanent teeth, and affected teeth are less resistant to friction but more resistant to acid etching (63, 64). Excessive fluoride intake can delay the mineralization of the enamel and increase its surface roughness, thus promoting plaque accumulation (65). Excessive fluoride binds more enamel amelogenin and increases enamel organic substances in the secretory phase of enamel formation, thereby leading to hypomineralization and DF (66, 67). The typical histological feature of DF is the low mineralization rate of the enamel, which becomes fragile, easily eroded, and exfoliated.

Dental fluorosis is rare and mild in deciduous teeth due to their development in the embryonic and lactation stages, and the placenta has a certain barrier effect on fluoride. However, if fluoride intake exceeds the limit of its screening function, it can also result in irregularities on the deciduous teeth (68).

In the literature included in this study, most studies only examined children for DF. The reported prevalence of DF was quite different between different studies, indicating that the heterogeneity of these studies may be strong. Although the prevalence of DF varied, all included studies (35, 36, 45–47) identified DF caused by brick tea as severe and of great concern.

### Relationship between SF and brick tea

Skeletal fluorosis is one of the most common and severe manifestations of fluorosis in adults, and it is also an important index to evaluate the severity of endemic fluorosis. Radiographic examination of SF may show increased bone matrix density and fibrous ossification, tendon attachment calcification, joint degeneration, degenerative hyperplasia, and ossification changes in the fibrous tissues (27, 69, 70). Such patients may also have DF, whereas those exposed to excess fluoride after 12 years of age may not have DF since the development of ameloblasts in the dental germ is finished by that age.

Cao et al. (27) indicated that the detection of SF positively correlated with long-term fluoride exposure from brick tea, and SF was increasing in individuals aged >50 years, while its severity was positively correlated with age.

Skeletal fluorosis needs to be differentiated from diseases such as osteoblastic metastatic carcinoma and renal bone disease. Patients with SF who experience pain should be given an appropriate dose of non-steroidal analgesics. Those with skeletal deformities should have local fixed or orthopedic surgery to prevent deformity exacerbation.

### Relationship between fluorosis and altitude

Previous studies (36, 45, 46) have shown that high-altitude is a risk factor for fluorosis. Animal experiments have shown that under low oxygen conditions, mice ingested more fluoride, and their enamel formation was affected. Higher altitudes result in a lower oxygen concentration of the air and lower pH levels of the urine, and they affect the metabolism of fluoride and calcium and increase the reabsorption of fluoride by the kidneys (71–73). Changes in acid–base balance caused by high-altitude hypoxia may increase fluoride levels in organisms’ internal environments by increasing the reabsorption of fluoride and reducing fluoride excretion in the urine.

The effects of altitude on the prevalence and severity of fluorosis were first reported (73) in Kenya. Rwenyonyi et al. (74) indicated that altitude was a risk indicator of fluorosis after controlling for confounding variables, such as water source and vegetarianism. An animal study (75) also indicated that rats in the high-altitude group had more severe cartilage damage, coagulative kidney necrosis, and hydropic liver degeneration than those in the low-altitude group.

**FIGURE 3:**
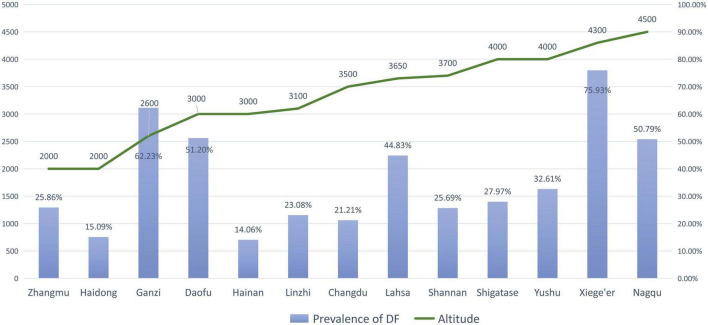
The prevalence of dental fluorosis (DF) and the altitude of surveyed areas (The line graph shows the altitude of the research site, and the bar graph shows the reported prevalence of DF at the research site).

**FIGURE 4:**
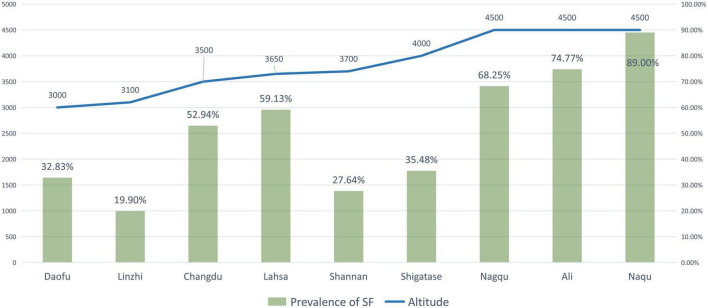
The prevalence of skeletal fluorosis (SF) and the altitude of surveyed areas (The line graph shows the altitude of the research site, and the bar graph shows the reported prevalence of SF at the research site).

Zhang et al. (46) reported that a higher altitude was a risk factor for DF, and the prevalence of DF at higher altitudes (2,560–3,300 m) was nearly three times higher than that at lower altitudes (1,400 m). When a study included districts with varying altitudes, the prevalence of DF in low-altitude areas tended to be lower than that in high-altitude areas (Table 3 and Figure 3). Similarly, this trend also existed for SF (Table 4 and Figure 4). However, these trends may not be obvious in the surveys of small samples, and they may be affected by various factors, such as the economic and health statuses of individuals living in the surveyed areas. In low-altitude areas, residents may have a more diverse diet, relatively better economic and hygiene conditions, and easier access to clean water sources. These confounding factors should be noted in future studies. Thus, large data with less heterogeneous results are needed to illustrate this issue.

### Relationship between BTF and occupation

Some researchers have proposed the possibility of correlations between BTF and residents’ occupation, economic status, and health. Fan et al. (45) analyzed the prevalence of fluorosis across different occupational populations in Tibet. For the rural population, occupations can be briefly divided into agriculture and animal husbandry. Interestingly, they indicated that the likelihood of BTF was significantly associated with occupational factors; it was higher in villages where animal husbandry was the main occupation, whereas it was lower in agricultural villages (45). They explained that, owing to harsh living conditions, herdsmen consumed less vegetables but more brick tea for vitamins and minerals, which might have led to a higher prevalence of fluorosis among them.

Similarly, the correlation between BTF and occupation may be associated with living conditions and economic factors. The correlation between BTF and occupation, like the correlation between BTF and altitude, may be merely an external manifestation of the correlations between BTF and socioeconomic and living conditions. More detailed studies need to be conducted to identify confounding factors for this issue.

Considering the influence of occupation on diet and ultimately the likelihood of fluorosis, the large number of part-time or full-time religious workers in Tibet cannot be ignored. Tibetan Buddhist religious people may choose to become a monk or nun for life or a period of time, and the food sources for them are limited owing to their religious beliefs. Zanba, butter tea, and brick tea constitute a high proportion of their diet; however, to date, no data on the prevalence of fluorosis exist for this group of people.

### Fluorosis and maternal brick tea consumption

During pregnancy and lactation, although the transfer of fluoride from mother to fetus is regulated by the placenta and the transfer of plasma to breast milk is inhibited (76), fluoride concentrations in breast milk and amniotic fluid increase significantly after the intake of fluorinated foods (77–81). Excessive fluoride exposure results in increased organic material in the secretory stage of enamel formation. Later, in the maturation period, the regulation of ameloblast modulation is disrupted by superfluous fluoride, and the mineralization of the enamel is disrupted, leading to hypomineralization and DF (66, 82, 83). Zhang et al. (46) reported an association between regular excessive maternal consumption of brick tea and DF in children. They indicated that the excessive consumption of brick tea by mothers during pregnancy and lactation could disrupt the development of children’s tooth germs.

In addition to the effect of fluoride levels on children’s teeth, existing research also suggests that higher levels of fluoride exposure in women during pregnancy may be associated with diseases or adverse reactions in their children. A report on fluoride intake in pregnant women indicated that those with a high intake produced offspring with lower IQs, and the effect was greater in boys (84). More attention should be paid to fluoride exposure in pregnant women.

### Relationship between genetic polymorphisms and BTF

Recent advancements in bioinformatics have revealed many diseases to be associated with a genetic predisposition (85). Fluorosis may result from excessive fluoride intake as well as from prolonged exposure and the body’s response, but epidemiological and animal studies (86–88) have also indicated individual differences in the severity of fluorosis.

It is speculated that fluorosis may be caused by the complex interaction between candidate genes of fluorosis and specific environmental exposure, including dietary fluoride intake, among others. The severity of fluorosis does not always depend on the consumption of fluoride. Genetic polymorphisms may lead to individual differences in sensitivity or resistance to fluoride exposure.

Existing studies have been conducted with different laboratory mouse strains to identify possible genetic determinants of DF. Everett et al. (89) showed that different genotypes of mice with the same fluoride levels could produce different degrees of DF severity when controlling for variables such as sex, food, age, housing, and drinking water fluoride concentration. Mousny et al. (90) analyzed the role of genetic factors on the effects of fluoride on bone metabolism in an animal study. Their study evaluated the effect of increasing fluoride doses in three inbred strains of mice (susceptible, resistant, and intermediate). Significant changes in bone quality were observed in susceptible strains, moderate changes were seen in intermediate strains, and no changes were noted in resistant strains. Thus, the findings suggest that genetic factors may contribute to variations in bone quality.

Some human studies have also confirmed the role of genetic factors in fluorosis. Researchers found that genes related to signal transduction (G-protein, ERK, MEK1, and MEK2) (91–93), immune lymphokines (interleukin 6, interleukin 8) (94, 95), and estrogen receptors (96) may influence the occurrence and development of fluorosis. Case-control studies (97–99) carried out in fluorosis-endemic areas have analyzed the relationship between gene polymorphisms of candidate genes and fluorosis susceptibility.

We found that many studies in China have analyzed the genetic diversity of BTF in Tibetan populations and evaluated the susceptibility to BTF caused by different genes in Tibetan, Kazak, Mongolian, and Han populations. GSTP1, vitamin D receptor-*Fok*I, prolactin promoter at -1,149, MMP2, FRZB1, serum soluble Klotho, BMP2, and ALOX15 and their related genes are thought to be involved (38, 49–55). However, the exact correlation between BTF and genetics needs further research.

### Preventive measures and effects of brick tea consumption

The prevention and control of BTF have been underway since the discovery of a link between brick tea consumption and fluoride intake in Tibetan areas. To reduce the effect of BTF, the government and disease control authorities have acted to rectify the production of brick tea containing high-fluoride concentrations. The standard in China (GB 19,965-2005) stipulates that the fluoride content of brick tea should not exceed 300 mg/kg. According to the classification standard of endemic fluorosis (GB 17,018-2011), an endemic fluorosis area can be determined if the average daily tea fluoride intake of people over 16 years of age is more than 3.5 mg and there are patients with SF confirmed by X-ray examination.

Strengthening health education allows residents to understand the harm of high-fluoride brick tea and consciously develop healthier tea drinking habits, such as changing the single diet structure, balancing nutrition, advocating for the reduced dependence on brick tea, not drinking high-fluoride tea, and eating more fresh vegetables and fruits.

In addition, the tea preparation method should be changed; before adding water to boil, the tea should first be mashed and washed once with 80°C water. Strong tea, tea that has been boiled for a long time, and tea that has been soaked for too long should be avoided. Tea drinkers should avoid adding alkali when boiling brick tea since the dissolution efficiency of fluoride in tea is higher under alkaline conditions.

Jin et al. (39) reported their findings from a 3-year (2008–2011) observation study on the prevention of brick tea-induced intoxication. By reducing the availability of brick tea in school canteens and switching to low-fluoride brick tea, the total daily fluoride intake of children decreased to a relatively safe level, although it was still at the level of chronic intoxication. Though the short-term use of low-fluoride brick tea reduced urinary fluoride levels, this difference was not significant, and the serum fluoride levels remained unchanged. This finding may have been attributed to the maintenance of internal homeostasis, and the bones may have become fluoride buffers in the body. Fluoride accumulation in the body may take a long time to be eliminated, and the effect of preventive measures on brick tea needs to be verified by controlled experiments with a larger sample over a longer period of time.

### Study limitations

To our knowledge, this is the first review of BTF in Tibetan residents, but limitations still exist. To date, the research on BTF is not comprehensive and has not been taken seriously enough. The literature that could be included in this study was limited, and most researches were conducted by the few limited scholars. In addition, the research methods are not unified and have a certain heterogeneity. The detection of fluoride in brick tea and the identification and rating of DF and SF should use widely recognized international standards. And no data are available on fluorosis among certain groups of Tibetans, especially Tibetan Buddhist monks and nuns. Lastly, the correlation between genotypes and the susceptibility to fluorosis caused by brick tea intake requires further investigation.

## Conclusion

At present, the high-fluoride content of brick tea is an important pathogenic factor for fluorosis in Tibetan regions. To our knowledge, this is the first review on the issue of BTF. DF had prevalence rates of 14.06–75.93% and an overall pooled rate of 26.08% in children, whereas SF had prevalence rates of 19.90–74.77% and an overall pooled rate of 33.84% in adults. BTF susceptibility may be associated with occupation and the altitude at which individuals reside. Genetic polymorphisms were suggested risk factors. BTF among Tibetan residents needs more attention, and further studies evaluating its pathogenesis and preventive measures are warranted.

## Author contributions

CW designed the study, applied for grant support, and conducted the literature search and screening, data collection, manuscript writing, and revision. QZ conducted the literature screening and data collection. FX participated in literature screening. JJ participated in data analysis. All authors read and approved the final manuscript.
