# Supplementary material for: Brick tea consumption and its relationship with fluorosis in Tibetan areas
Source: Front Nutr. 2022 Dec 13;9:1030344. doi: 10.3389/fnut.2022.1030344 (PMC9792988; doi:10.3389/fnut.2022.1030344)
Supplement: Supplementary file 1 [file Table_1.docx]

**PRISMA Flow Diagram**

## Identification

## Screening

Records identified through database searching
(n = 297 )

Records after duplicates removed
(n = 297 )

Records excluded
(n = 274)

Records screened
(n =297 )

## Eligibility

Full-text articles excluded, with reasons
(n = 8)

Full-text articles assessed for eligibility
(n = 23 )

## Included

1.Group of prevalence of BTF (n=6)^*^

2. Group of fluoride content of brick tea (n=4)^*^

3.Group of Genetic relationship with BTF (n=8)

Studies included in brick tea-type fluorosis in different groups (n=15)

* 3 articles are included in both group 1 and 2
